# IL1A regulates the inflammation in gout through the Toll-like receptors pathway

**DOI:** 10.7150/ijms.88447

**Published:** 2024-01-01

**Authors:** Meirong Ling, Jiaqi Gan, Mengting Hu, Fei Pan, Mei Liu

**Affiliations:** 1 Emergency Medical Department, Minhang Hospital, Fudan University, 170 Xinsong Road, 201199, Shanghai, China.; 2 Department of General Medicine, Minhang Hospital, Fudan University, 170 Xinsong Road, 201199, Shanghai, China.

**Keywords:** Gout, Monosodium urate, GSE199950, WGCNA, IL1A, Toll like receptor signaling pathway

## Abstract

**Objective:** Gout is a dangerous metabolic condition related to monosodium urate (MSU). Our aim is to study the molecular mechanisms underlying gout and to identify potential clinical biomarkers by bioinformatics analysis and experimental validation.

**Methods:** In this study, we retrieved the overlapping genes between GSE199950-Differential Expressed Genes (DEGs) dataset and key module in Weighted Gene Co-Expression Network Analysis (WGCNA) on GSE199950. These genes were then analyzed by protein-protein interaction (PPI) network, expression and Gene Set Enrichment Analysis to identify the hub gene related to gout. Then, the gene was investigated by peripheral blood mononuclear cells (PBMCs), immunoassay and cell experiments like western blotting to uncover its underlying mechanism in gout cells.

**Results:** From the turquoise module and 83 DEGs, we identified 62 overlapping genes, only 11 genes had mutual interactions in PPI network and these genes were highly expressed in MSU-treated samples. Then, it was found that the IL1A (interleukin 1 alpha) was the only one gene related to Toll-like receptor signaling pathway that was associated with the occurrence of gout. Thus, IL1A was determined as the hub gene in this study. In immunoassay, IL1A was significantly positively correlated with B cells and negatively correlated with macrophages. Moreover, IL1A is highly expressed in gout patients,it has a good clinical diagnostic value. Finally, the results of *in vitro* experiments showed that after knocking down IL1A, the expressions of pro-inflammatory cytokines and Toll-like receptor signaling pathway-related proteins (TLR2, TLR4, MyD88) were all reduced.

**Conclusion:** It is confirmed that IL1A is a promoting gene in gout with a good diagnostic value, and specifically it affects the inflammation in gout through Toll-like receptor pathway. Our research offers fresh perspectives on the pathophysiology of gout and valuable directions for future diagnosis and treatment.

## Background

Gout, a chronic inflammatory arthritis disease, is characterized by hyperuricemia and caused by interactions between genetic, epigenetic, and metabolic factors^1^. Gout is a dangerous metabolic condition that is directly connected to hyperuricemia caused by purine metabolism abnormalities or reduced uric acid excretion^2^. According to research, type Ia glycogen storage disease, Lesch-Nyhan syndrome, chronic kidney disease all can also lead to abnormal uric acid metabolism, which initiates the development of gout^3^-^5^. In addition, thiazide diuretics, cyclosporine, warfarin and other drugs, purine-rich food intake, excessive alcohol consumption, obesity, diabetes, cardiovascular disease, and other factors are the main triggering factors of gout^6^, ^7^. Gout is an autoimmune disease associated with localized immune attacks. Immune cells are associated with or related to the immune response, which primarily includes asymptomatic hyperuricemia, acute gouty arthritis, critical gout, and chronic gout^8^. The most noticeable symptom is abrupt mild pain in one or more joints, which is accompanied by joint redness and swelling, as well as an increased skin temperature^9^, ^10^. As a result, it is important to minimize acute joint inflammation as quickly as possible, lower the blood uric acid level, and avoid the development of uric acid nodules during the treatment of gout.

Monosodium urate (MSU) is considered to be related to the pathogenesis of gouty arthropathy and the formation of calcium oxalate stones^11^. It is soluble in water, almost insoluble in ethanol, and the liquid is alkaline when MSU is dissolved in water. 12. MSU crystals are influenced by temperature, concentration, dissolution and excretion^13^. When the concentration of MSU rises to saturation, crystals precipitate in cavities such as joints, which gradually enlarge with decreased temperature, leading to the development of gout^14^. And MSU provokes the activation of innate immune system and thereby, elicits strong inflammatory response in the joint and periarticular tissues^15^, ^16^. Infiltrated macrophages engulf MSU via phagocytosis and subsequently release an array of inflammatory mediators^17^, some of these mediators may possibly interact with the peripheral nociceptors to elicit gout pain^18^.At present, relevant experiments have shown that MSU can induce acute gouty arthritis in rodents, and MSU crystals can also affect the activity and function of gouty arthritis-related cells^19^, ^20^. Based on this, we can search for MSU-related data in public databases to explore key biomarkers in the pathogenesis of gout.

We investigated the GSE199950 dataset associated with MSU in the Gene Expression Omnibus (GEO) database. Through the bioinformatics analysis on samples in this dataset, hub gene (IL1A) and key pathway (Toll like receptor signaling pathway) related to gout were identified. Besides,* in vitro* assays were performed on IL1A to investigate the potential link between IL1A and pro-inflammatory cytokines. This study will offer new perspectives on the molecular mechanism of gout.

## Material and methods

### PBMC Harvesting

Peripheral blood mononuclear cells (PBMC) were obtained by density gradient centrifugation from blood samples of 10 primary gout patients as well as 5 healthy controls (HC), respectively, and stored at -80°C. The mRNA expression level of IL1A in PBMC was determined by quantitative reverse transcription-polymerase chain reaction. Patient characteristics are shown in Supplementary Table 1. This study was approved by the Ethics Committee of the Minhang Hospital, Fudan University and was registered with the Committee in accordance with protocol number 009-01X.

### Establishment of gene co-expression network

The GSE199950 dataset contains data related to gout, with a total of 6 samples and 27281 genes. We built a gene co-expression network by these genes by the method of Weighted gene co-expression network analysis (WGCNA), and set the optimal soft threshold. The adjacency matrix was changed into a topological overlap matrix (TOM), the genes were classified into several gene modules based on the dissimilarity of the TOM, thus Cluster Dendrogram and Eigengene adjacency heatmap were produced. Then, the correlation and significance between the modules and the sample features were calculated to determine the key module.

### Analysis of the key module

The Gene Ontology (GO) term includes three branches, biological process (BP), cell component (CC), molecular function (MF), which can clearly understand which genes may be related to the changes in gene functions^21^. Kyoto Encyclopedia of Gene and Genome (KEGG) enrichment analysis can help researchers better understand the biological pathways in which genes are involved^22^. After obtaining the key module, we conducted GO term and KEGG pathway enrichment analysis on the genes in the module with the help of the "Cluster Profiler" package of the R software. After that, we uploaded the gene information from the key module to the Cytoscape software and constructed a Protein-Protein Interaction (PPI) network.

### The identification of GSE199950-DEGs

In the 6 samples of the GSE199950 dataset, lymphatic endothelial cells (LECs) with Phosphate Buffered Saline (PBS) was set as the control group, and LECs treated with 300μg/ml MSU as the case group. Then, based on the GEO2R tool, FC>2 was for up-regulation, FC<0.5 for down-regulation, both *P*<0.05. Then, DEGs were subjected to KEGG enrichment analysis using the Gene Set Enrichment Analysis (GSEA) website.

### The Search Tool for the Retrieval of Interacting Genes (STRING) database

With the help of the "VennDiagram" package, we obtained the overlapping genes from the key module and GSE199950-DEGs. The data from these genes were then submitted to STRING database (https://string-db.org/) to build a PPI network in order to search for the interaction between these genes. Subsequently, the PPI network was visualized by the Cytoscape tool, and 11 genes with tight interaction were obtained. Next, the levels of these key genes in GSE199950 samples were verified, and corresponding boxplots were drawn for analysis. Moreover, the KEGG pathway enrichment analysis was conducted on 11 key genes through the GSEA software, and the enrichment results with a significant *P*<0.05 were found. Then, the gene enriched in the gout-related pathways was screened out and regarded as the hub gene for further analysis.

### Infiltration analysis between the hub gene and immune cells and Receiver operating characteristic (ROC) analysis

A program called SangerBox (http://vip.sangerbox.com/login.html) has a number of biological information analysis, visual mapping, and practical data download features^23^. In order to explore the biological characteristics of the hub gene, we verified the correlation between the hub gene and 24 kinds of immune cells on this website, calculated the correlation coefficient, and screened the significant results with *P*<0.05. Then, ROC curve of the hub gene was drawn by the "timeROC" package, and the Area Under Curve (AUC) value and confidence interval (CI) were calculated to evaluate the clinical diagnostic value of the hub gene.

### Cell culture and transfection

The human THP-1 monocytes were acquired from the American Type Culture Collection and cultured in RPMI 1640 medium with 10% FBS at 37ºC in a 5% CO_2_ incubator. Invitrogen provided the IL1A small interfering RNA (siRNA) and the negative control (NC). Following the manufacturer's instructions, siRNAs were transiently transfected into the THP-1 cell line using Lipofectamine 3000 reagent (Invitrogen).

### Real-time quantitative PCR (qRT-PCR) assay

TRIzol reagent (Cat. 15596026, Invitrogen) was used to extract total RNA. Reverse-transcribe total RNA (2 g) into complementary DNA (cDNA) for mRNA assays by using a first-strand cDNA synthesis kit (Cat. 11119ES60, Yeasen), Subsequently, THP-1 cells induced with different concentrations of MSU (0 μg/ml, 50 μg/ml and 100 μg/ml) 24, using the SYBR Green qPCR Kit (Cat. 11203ES03) to detect the concentrations of IL1A and three pro-inflammatory cytokines (IL-8, IL-1β, TNFα), qRT-PCR analysis was carried out on a TL998-IV Real-Time PCR System. As an internal control, GAPDH was used. The 2^-ΔΔCt^ method was used to examine the findings of qRT-PCR. The primers sequences were as follows: IL1A-forward, 5'-ATCATGTAAGCTATGGCCCACT-3′, IL1A-reverse, 5'-CTTCCCGTTGGTTGCTACTAC-3′; IL-1β-forward, 5'-TCTGGTAATCCACTCAAATAGGGA, IL-1β-reverse, 5'-ACCTTGTGATGTAGTGTTGTGGT-3′; IL-8-forward, 5'-TGAGCAGATCTTGCATGTAGC-3′, IL-8-reverse, 5′-GCTGCTGATCAAGAAGATTCACC-3′; TNFα-forward, 5′-GGACACTGGCCACTTACGAA-3′, TNFα-reverse, 5′-GCCAACCTTCCCAAAACCAC-3′; TLR2-forward, 5′-AATTGTGACCAAGACGGGACA-3′, TLR2-reverse, 5′-GTTGACTGGTGAGCGACGA-3′; TLR4-forward, 5'-TTGTGCAAACTTGCCGGGAGGA-3', TLR4-reverse, 5'-ACTTCTCCTTCAGCTTGGCAGC-3'; MyD88-forward, 5'-CCAACGCCAGCAAAGTTCTC-3', MyD88-reverse, 5'-AGGTCCACACAAAACCCCTG-3'; GAPDH-forward, 5′-GCACCGTCAAGGCTGAGAAC-3′, GAPDH-reverse, 5′-TGGTGAAGACGCCAGTGGA-3′.

### Western blotting (WB) assay

RIPA Lysis Buffer extracts total protein from cells, and a Bio-Rad Protein Assay Kit (Bio-Rad Laboratories) was used to assess these protein quantities. SDS-PAGE was used to separate the proteins, and the membranes were PVDF. Following that, they were blocked with 5% skim milk and incubated with primary antibodies (IL1A, IL-1β, IL-8, TNFα, TLR2, TLR4, MyD88) for 2 hours indoor. Subsequently, the secondary antibody (conjugated goat anti rabbit IgG) was treated with the membrane for two hours at room temperature, and the results were visualized using an ECL kit (ECL Substrate Kit, ab133406, Abcam). GAPHD was used as an internal reference, and all steps were repeated more than three times. ImageJ software was used to quantify the gray scale of the protein bands.

### Statistical analysis

For statistical analysis, the SPSS 22.0 program was employed. The data were expressed as mean±standard deviation. One-way ANOVA was applied when comparing more than two patient groups. *P* value less than 0.05 was used to establish a statistically significant difference.

## Results

### The turquoise module was the key module in the gene co-expression network

**Figure 1:**
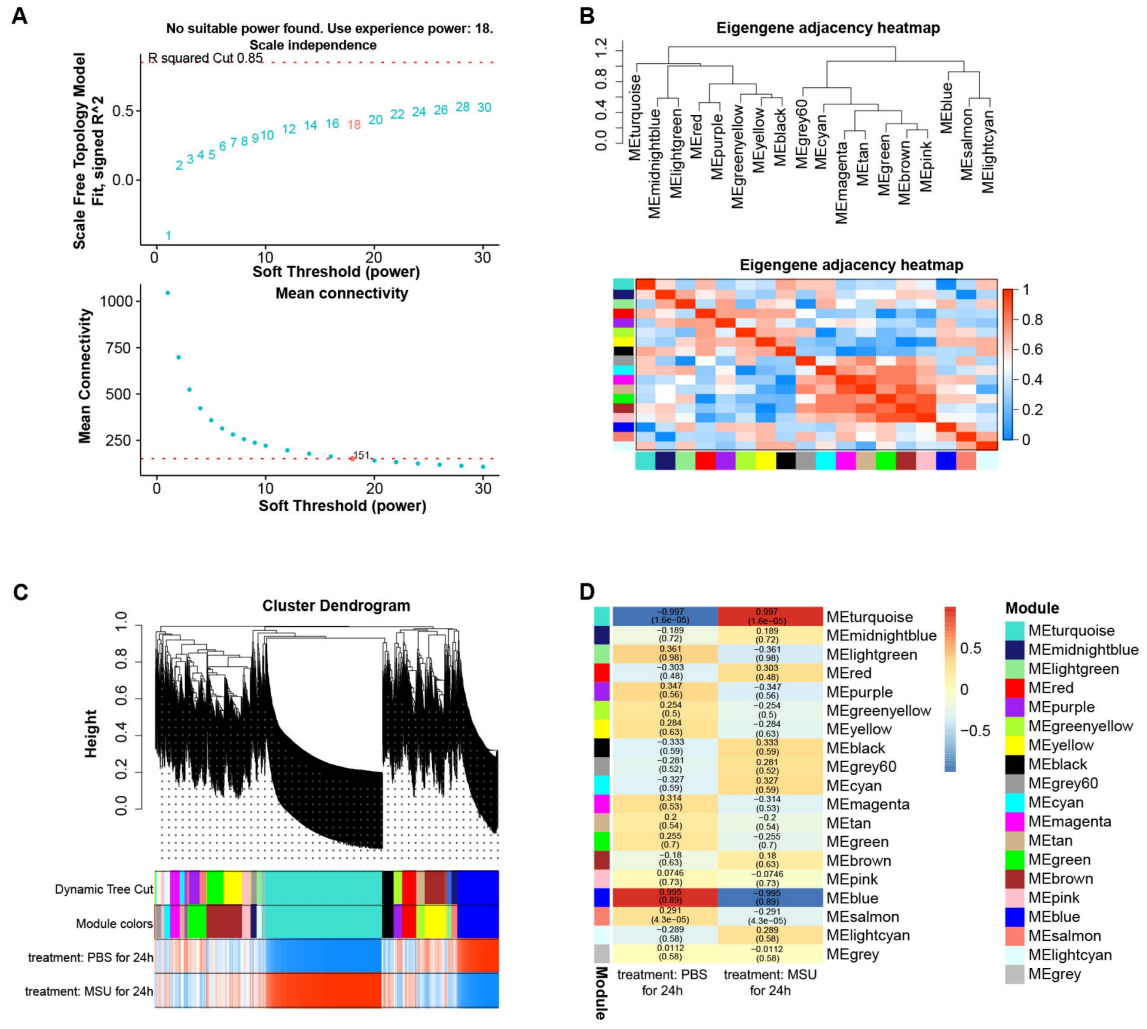
**The turquoise module was the key module in the gene co-expression network.** (A) The above figure is a reference for the optimal soft threshold, and the red line represents the subjectively selected scale-free fitting index value. The graph below shows the mean connectivity at different soft thresholds. (B) Eigengene adjacency heatmap. The top is a dendrogram of different color modules, and the bottom is a clustering heat map of genes in different modules. The redder the color, the higher the correlation. (C) Cluster dendrogram of gene modules, a total of 18 modules were clustered after merge. (D) Heatmap of the relationship between gene modules-features. Columns represent different samples of GSDE199950 and rows represent gene modules.

By WGCNA method, we built a gene co-expression network with an optimal soft threshold of 18 based on 27,281 genes in the GSE199950 dataset (Figure 1A). Then, we divided genes into 18 modules with different colors according to the dissimilarity of TOM, (the grey module belonged to invalid module, no analysis was performed) (Figure 1B). The correlations of these modules were all less than 0.8, and there was no need to merge the modules. The cluster dendrogram of the 18 module assignments and the heatmap of the positive (red) or negative (blue) correlations with the two groups of samples in the GSE199950 dataset were presented in Figure 1C. Subsequently, by computing the correlation between the module and the sample features, it was found that the turquoise module (PBS for 24h: r=-0.997, *P*=1.6e-05; MSU for 24h: r=0.997, *P*=1.6e-05) had the strongest correlation with the GSE199950 sample (Figure 1D). Thus, the turquoise was considered the key module.

### Enrichment analysis and PPI network of the turquoise module

**Figure 2:**
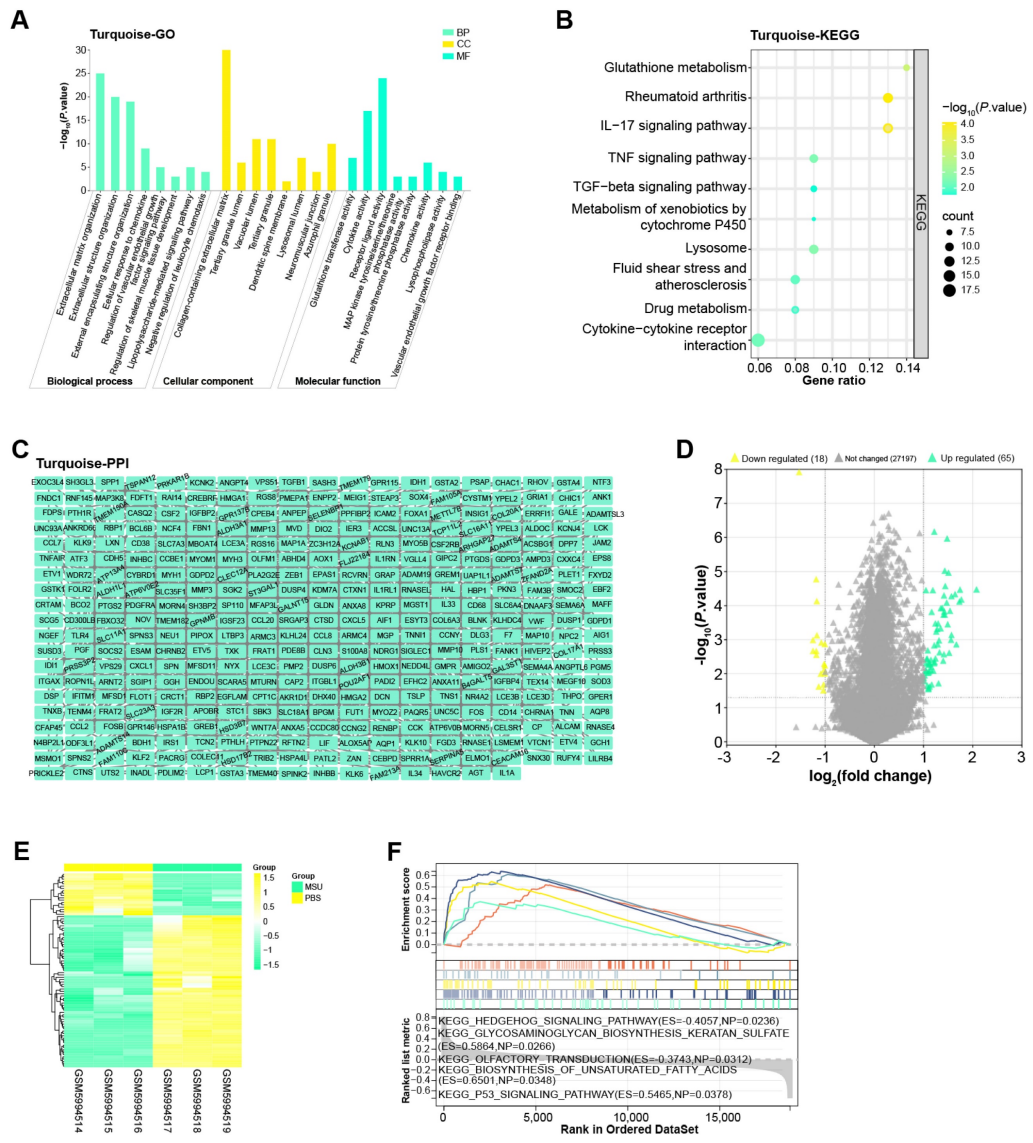
** Enrichment analysis and PPI network of the turquoise module.** (A) Histogram of GO enrichment analysis of all genes in the turquoise module. (B) Bubble plot of KEGG pathway enrichment analysis of all genes in the turquoise module. The abscissa indicates the gene ratio, and the ordinate indicates the enriched KEGG pathway. (C) PPI network of genes in the turquoise module constructed based on Cytoscape. (D) Volcano plot of GSE199950-DEGs, yellow scatter on the left indicates down-regulated DEGs, and green scatter on the right indicates up-regulated DEGs. (E) Heatmap of GSE199950-DEGs, green column indicates MSU group and yellow column indicates PBS group. (F) GSEA-KEGG enrichment analysis of GSE199950-DEGs.

The turquoise module included a total of 691 genes, for which we performed enrichment analysis (Figures 2A and 2B). Among them, in the BP results of the GO term, the enrichment items of these genes were extracellular matrix organization, regulation of vascular endothelial growth factor signaling pathway and so on. In CC, Collagen-containing extracellular matrix, Tertiary granule lumen, Vacuolar lumen, etc. were also associated with genes in the turquoise module. In MF, these genes were also related to cytokine activity, receptor ligand activity, etc. Moreover, KEGG also showed that the top 10 pathways enriched in these genes were glutathione metabolism, TGF-beta signaling pathway, fluid shear stress and atherosclerosis, drug metabolism, cytokine-cytokine receptor interaction. Subsequently, Cytoscape software also constructed the PPI network of these genes, including 377 nodes and 928 edges (Figure 2C).

### Functional enrichment analysis on GSE199950-DEGs

By the screening criteria of GEO2R, we obtained 65 up-regulated and 18 down-regulated DEGs, which were shown in Figures 2D and 2E. Next, the first five KEGG pathways of these DEGs were hedgehog signaling pathway, glycosaminoglycan biosynthesis keratan sulfate, olfactory transduction, biosynthesis of unsaturated fatty acids, and P53 signaling pathway (Figure 2F).

### The 11 potential hub genes identified from turquoise module and GSE199950-DEGs

**Figure 3:**
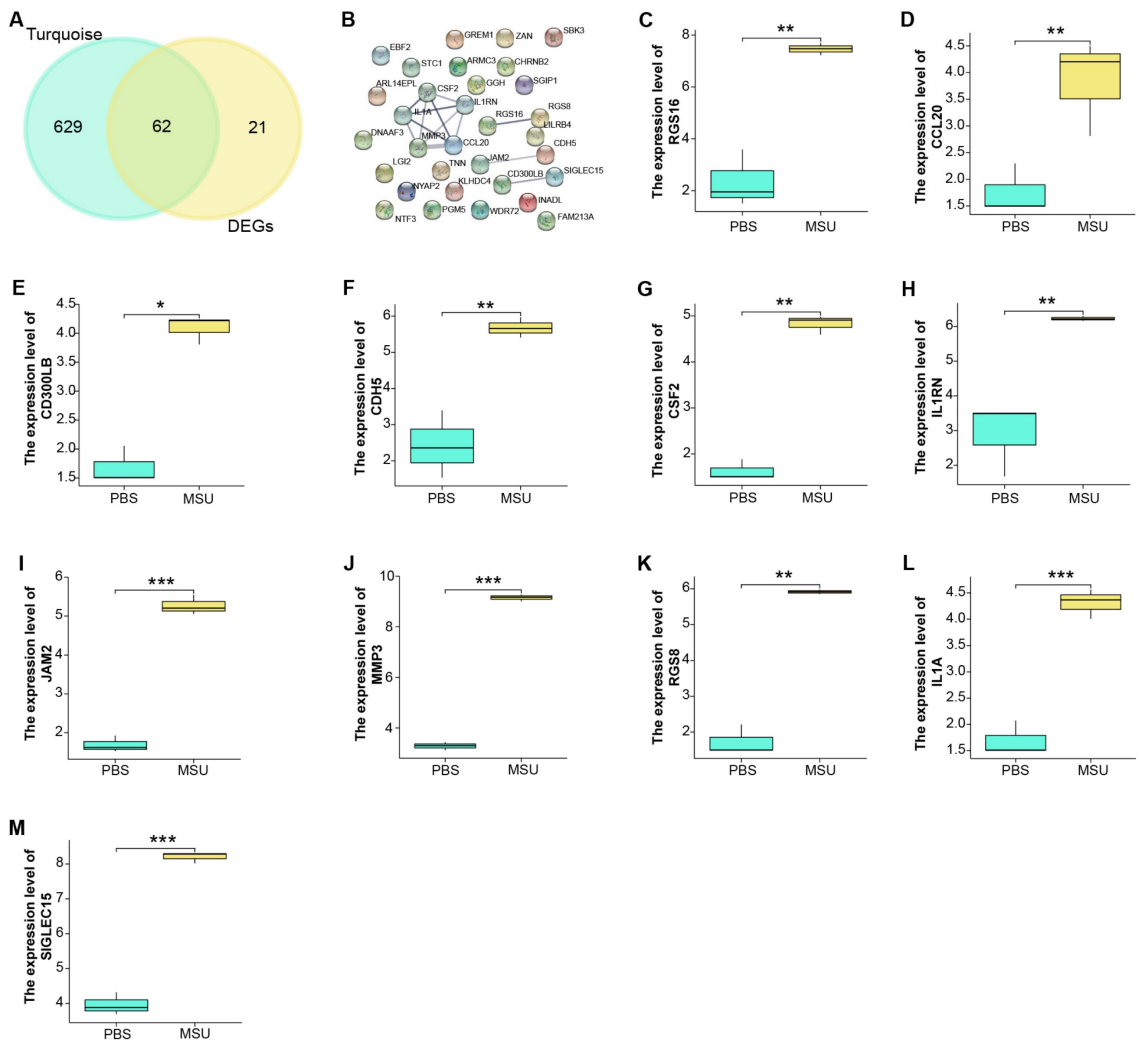
**Functional enrichment analysis on GSE199950-DEGs.** (A) Venn diagram, 62 genes overlap between the genes in the turquoise module and GSE199950-DEG. (B) PPI network constructed from the STRING database, where nodes represent genes and edges represent the interconnections between genes. (C-M) Expression verification of the genes corresponding to 11 nodes in the control and case groups of GSE199960, respectively RGS16, CCL20, CD300LB, CDH5, CSF2, IL1RN, JAM2, MMP3, RGS8, IL1A, SIGLEC15. Control group:lymphatic endothelial cells (LECs) with Phosphate Buffered Saline (PBS); Case group:LECs treated with 300μg/ml MSU. *P<0.05, **P<0.01, ***P<0.001.

We identified a total of 62 overlapping genes from 691 genes in the turquoise module and 83 DEGs in GSE199950 (Figure 3A). Then, the interaction between these genes was analyzed through the STRING database to obtain a PPI network (Figure 3B). Among them, there were 11 closely related genes, namely MMP3, RGS16, CDH5, CSF2, CD300LB, IL1RN, JAM2, SIGLEC15, RGS8, IL1A, CCL20. Subsequently, we detected the levels of these 11 genes in the GSE199950 samples, and from the results in Figures 3C-3M, these 11 genes were generally increased in the case group.

### IL1A was the hub gene identified by GSEA

**Figure 4:**
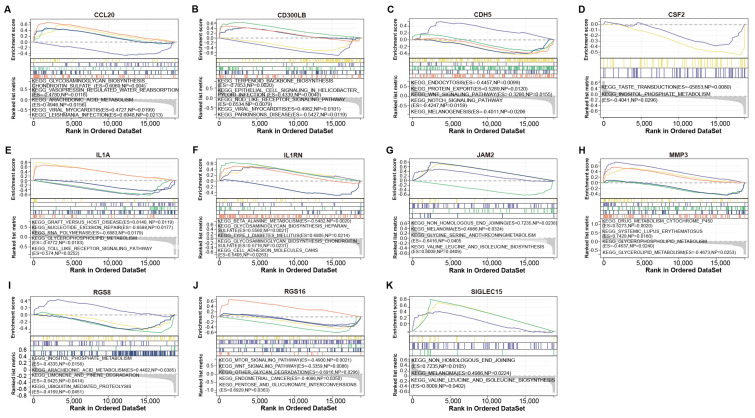
**GSEA-KEGG enrichment analysis of 11 genes.** (A) CCL20. (B) CD300LB. (C) CDH5. (D) CSF2. (E) IL1A. (F) IL1RN. (G) JAM2. (H) MMP3. (I) RGS8. (J) RGS16. (K) SIGLEC15. The different colored lines correspond to the signaling pathways below as well as positive and negative correlations and significance.

To verify the biological functions of 11 genes, we performed GSEA-KEGG pathway enrichment analysis on these genes (Figures 4A-4K). These 11 gene-enriched pathways included glycosaminoglycan biosynthesis chondroitin sulfate, vasopressin regulated water reabsorption, viral myocarditis, taste transduction, RNA polymerase,Toll-like receptor signaling pathway, beta alanine metabolism, inositol phosphate metabolism, MTOR signaling pathway, melanoma, etc. Among them, the Toll-like receptor signaling pathway enriched by IL1A is an inflammatory pathway, which is related to gout, so we targeted IL1A for subsequent analysis.

### IL1A had a good diagnostic value in gout

**Figure 5:**
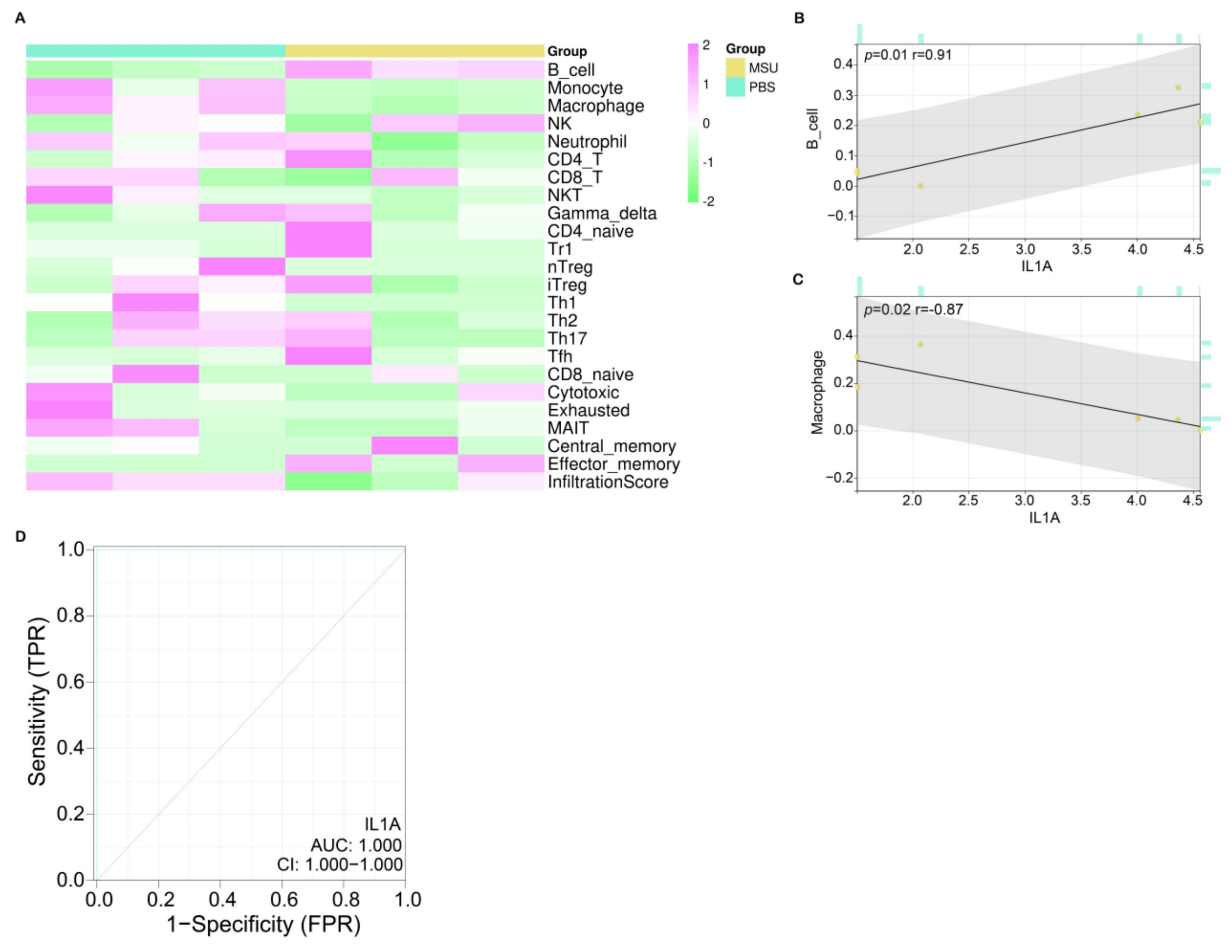
** Immunoassay and ROC curve analysis of IL1A.** (A) Heat map, turquoise column indicates PBS-treated group, ginger yellow column indicates MSU-treated group, each row indicates one immune cell. (B) Correlation analysis between IL1A expression and B cell infiltration level, the upper left corner shows the *P* value and the correlation coefficient r value. (C) Correlation analysis between IL1A expression and Macrophage infiltration level, the upper left corner shows the *P* value and the correlation coefficient r value. (D) The ROC curve of the prognostic value of IL1A, the abscissa is 1-Specificity (False Positive Rate; FPR), the ordinate is the sensitivity (True Positive Rate; TPR), the larger the AUC value, the higher the prediction accuracy.

After screening the hub gene, we first verified the correlation between 24 immune cells in the ImmuneCellAI database and IL1A based on the Sangerbox tool (Figure 5A). According to the results in Figures 5C and 5D, two types of cells were found to be significantly correlated with IL1A, namely B cell (*P*=0.01) and Macrophage (*P*=0.02). Among them, the expression of IL1A was positively related to the infiltration of B cell, and the correlation coefficient r was 0.91. On the contrary, the expression of IL1A was negatively correlated with the infiltration level of Macrophage, and the correlation coefficient r was -0.87. Then, according to the ROC curve, we calculated the AUC value of IL1A to be 1.000 and the CI to be 1.000-1.000, indicating a good diagnostic ability for the gout patients (Figure 5D).

### Downregulation of IL1A expression reduced the expression of pro-inflammatory cytokines

**Figure 6:**
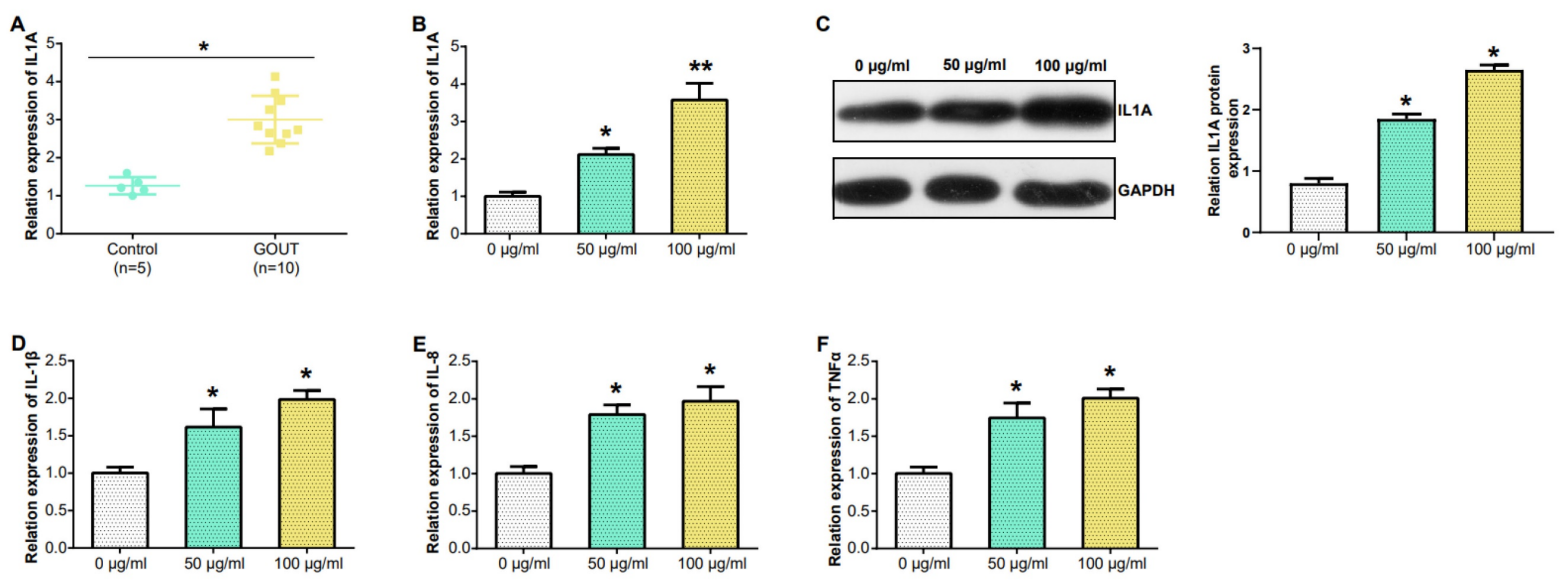
**Expression of IL1A in PBMC and MSU concentration in THP-1 cells detected by qRT-PCR.** (A) IL1A mRNA level in the PBMC from patients (n = 10), and HC (n = 5) were detected by qRT-PCR. (B) qRT-PCR detection of different concentrations of MSU induced the level of IL1A mRNA in THP-1 cells. (C) The level of IL1A protein in THP-1 cells induced by different concentrations of MSU was detected by WB. (D-F) The mRNA levels of IL-1β, IL-8 and TNFα in THP-1 cells induced by different MSU concentrations were detected by qRT-PCR. *P<0.05, **P<0.01.

**Figure 7:**
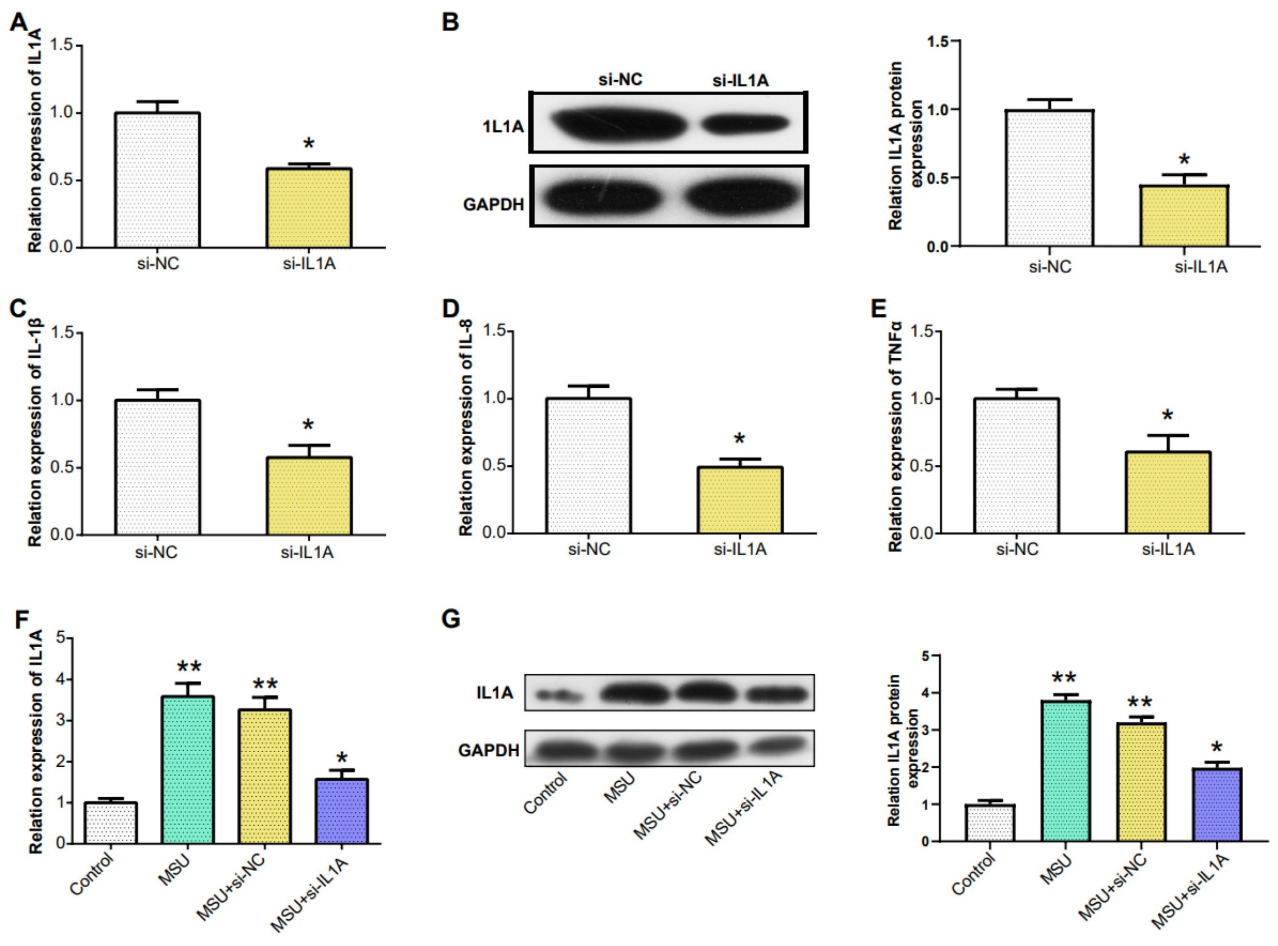
**Downregulation of IL1A expression reduced the expression of pro-inflammatory cytokines.** (A) qRT-PCR detection of MSU-induced knockdown efficiency of IL1A in THP-1 cells. (B) The knockdown efficiency of IL1A in THP-1 cells induced by MSU was detected by WB. (C-E) qRT-PCR was used to detect the mRNA expressions of IL-1β, IL-8, and TNFα in MSU-induced THP-1 cells after knocking down IL1A. (F) qRT-PCR was used to detect the mRNA expressions of MSU, MSU+si-NC, MSU+si-IL1A. (G) The protein expressions of MSU, MSU+si-NC, MSU+si-IL1A were detected by WB. *P<0.05, **P<0.01.

In PBMCs, IL1A levels in gout patients were higher compared to HCs (Figure 6A). From the results in Figure 6B, the IL1A level inTHP-1cells induced by 100 μg/ml MSU concentration was higher than that induced by 50 μg/ml MSU concentration. This was also confirmed in the WB results and quantify the gray scale of the protein bands (Figure 6C). qRT-PCR also detected that after THP-1 cells were induced with 100 μg/ml MSU concentration, the level of three pro-inflammatory cytokines (IL-1β, IL-8, TNFα) was also up-regulated (Figures 6D-6F). Subsequently, we firstly treated THP-1 cells with 100 μg/ml MSU concentration, and knocked down IL1A. qRT-PCR and Western blotting analyses, along with gray-scale evaluation of protein levels, revealed a significant reduction in both mRNA and protein expressions of IL1A (Figures 7A and 7B). In addition, as the level of IL1A was reduced, the levels of IL-8, IL-1β, and TNFα also decreased (Figures 7C-7E). The outcomes gleaned from qRT-PCR and WB analyses, complemented by protein grayscale quantification, revealed significant modulations in IL1A expression in response to MSU exposure and a subsequent intervention with MSU combined with si-IL1A. Specifically, there was an upregulation of IL1A expression following treatment with MSU, an effect that was notably attenuated upon the introduction of si-IL1A in the MSU treatment protocol, as evidenced when benchmarked against the control (Figures 7F-7G). Therefore, we concluded that IL1A positively regulates the expression of pro-inflammatory cytokines.

### IL1A regulates the inflammatory response in gout via Toll-like receptor signaling pathway

**Figure 8:**
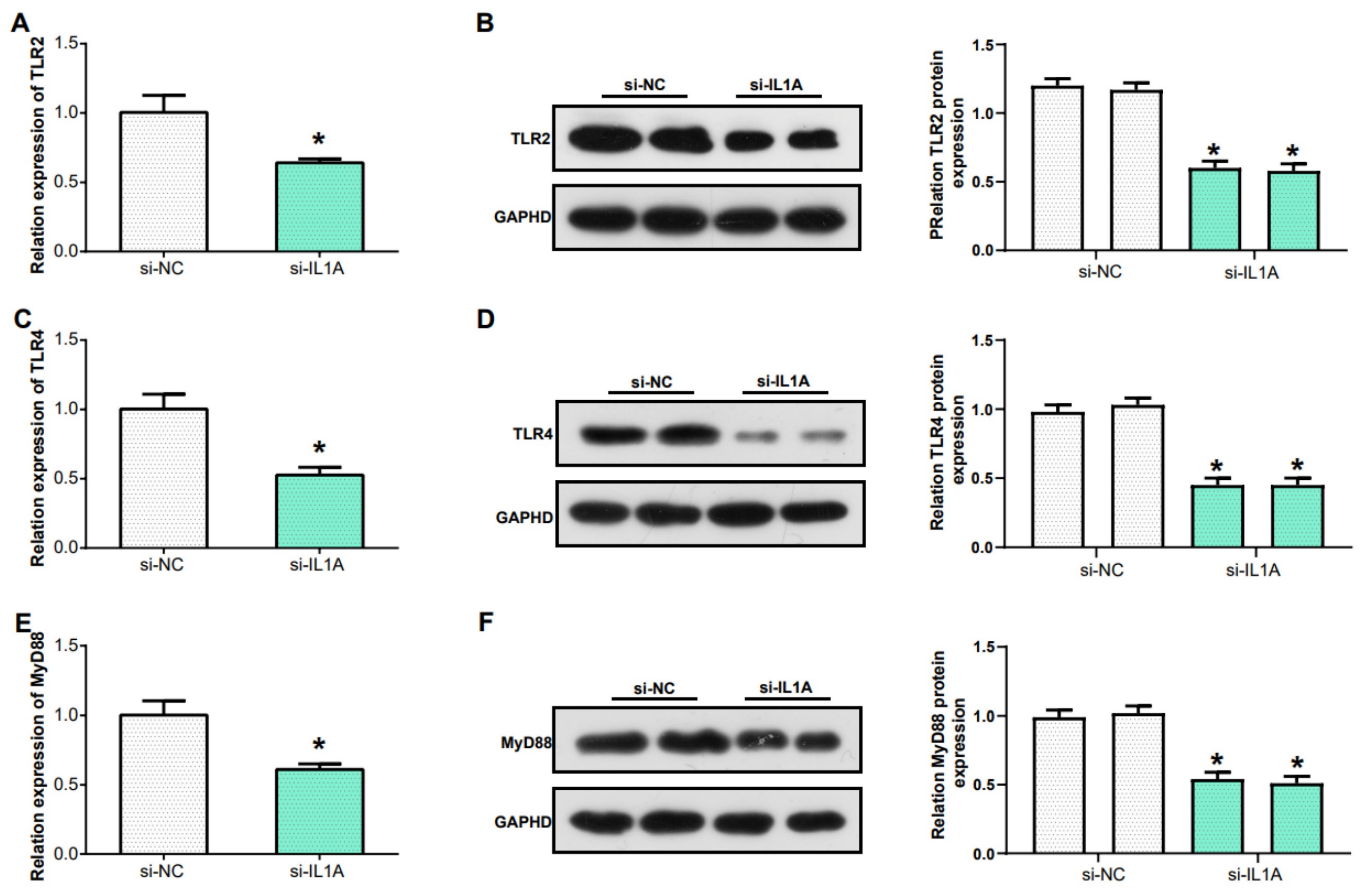
** IL1A regulates the inflammatory response in gout via Toll-like receptor signaling pathway.** (A, C, E) qRT-PCR was used to detect the mRNA expression of TLR2, TLR4 and MyD88 after IL1A knockdown in THP-1 cells induced by MSU. (B, D, F) WB assay for protein expression of TLR2, TLR4 and MyD88 after knockdown of IL1A in MSU-induced THP-1 cells. *P<0.05.

In the previous research Toll-like receptor signaling pathway was identified as the key pathway in gout. TLR2 and TLR4, two members of the Toll-like receptor family, play a critical role in recognizing different types of pathogens^25^. MyD88 acts as an adaptor protein closely associated with the Toll-like receptor signaling pathway, and it is a key molecule in this signaling transduction pathway^26^. In this study, we examined the levels of TLR4, TLR2, and MyD88 in THP-1 cells after knockdown of IL1A expression. The mRNA levels of TLR2, TLR4 and MyD88 were downregulated (Figures 8A, 8C and 8E). Similarly, WB and protein grayscale quantification also indicated the protein levels of TLR2, TLR4 and MyD88 were down-regulated (Figures 8B, 8D and 8F). Therefore, we conclude that IL1A may participate in the inflammation of gout through positively regulating Toll like receptor signaling pathway.

## Discussion

The recurring inflammatory condition known as gout is brought on by an increase in purine biosynthesis and metabolism, an excess generation of uric acid, or a deficiency in uric acid excretion, which results in an accumulation of urate crystals in the blood, more common in medium-elderly men^27^, ^28^. This condition is characterized by acute arthritis, tophi, interstitial nephritis, joint deformity, and dysfunction^29^, ^30^, often accompanied by uric acid urolithiasis^31^. As the incidence of gout continues to rise, it can induce other symptoms, such as hyperglycemia, hyperlipidemia, kidney damage, and physical disability^32^, ^33^. Existing studies have confirmed that MSU is related to the pathogenesis of gouty arthropathy as one of the incentives for gout^34^. Therefore, we retrieved the GSE199950 dataset related to gout and MSU, and performed multiple bioinformatics analyses on this dataset to find key biomarkers in the pathogenesis of gout.

Herein, we employed the WGCNA algorithm to build a gene co-expression network with 27,281 genes from the GSE199950 dataset. Through the analysis, it was found that the relationship between the turquoise module and the GSE199950 samples was the strongest, and then it was considered the key module. The genes in the key module were mainly related to Extracellular matrix organization, Glutathione metabolism, Rheumatoid arthritis, IL-17 signaling pathway, etc. Then, 83 DEGs were analyzed from the GSE199950 dataset, mainly related to Hedgehog signaling pathway, Olfactory transduction, and P53 signaling pathway, etc. Among the enriched items of the above genes, some have been confirmed to be involved in the pathogenesis of gout.

For example, Liu P et al. identified the IL-17 signaling pathway as a key pathway related to gout treatment through network pharmacology analysis^35^. Chen F et al. discovered that some long noncoding RNAs (lncRNAs) could compete with mRNAs for microRNAs (miRNAs), affect the IL-17 and TNF signaling pathways by cell response to chemical stress, thus contributing to gout pathogenesis^36^. Fan Y et al. also mentions that the P53 signalling pathway mediates the therapeutic process of compound Si Miao San in acute gouty arthritis^37^.

In the following study, 62 overlapping genes were identified from 691 genes in the turquoise module and 83 DEGs in the GSE199950 dataset. Subsequently, the PPI network of these genes was established by Cytoscape, and 11 genes were closely connected, namely CCL20, CD300LB, CDH5, CSF2, IL1A, IL1RN, JAM2, MMP3, RGS8, RGS16, SIGLEC15. Moreover, the results of expression verification indicated 11 genes were up-regulated in the samples treated with MSU. In order to continue to study the biological characteristics of these genes, we analyzed the enrichment pathways of these genes in KEGG based on GSEA, and obtained Glycosaminoglycan biosynthesis chondroitin sulfate, Viral myocarditis, Taste transduction, RNA polymerase, Toll-like receptor signaling pathway, Beta alanine metabolism, Melanoma, etc. Among them, the Toll-like receptor signaling pathway enriched by IL1A gene is an inflammation-related pathway, which has been confirmed to be involved in the pathogenesis of gout^38^. The toll-like receptor 4-NFκB-IL1β signaling pathway mentioned in the article by Qing YF et al. is associated with changes of peripheral blood in gout patients, which can affect the development of primary gouty arthritis^39^. Therefore, we suspected IL1A regulated Toll-like receptor signaling pathway to participate in the pathogenesis of gout.

A member of the interleukin-1 cytokine family, IL1A is a pleiotropic cytokine implicated in a variety of immunological responses, inflammatory processes, and hematological activities^40^. When a cell is injured, this gene is generated by macrophages as a pre-protein, which is then processed by proteolysis and released, causing cell death^41^. Some data also suggest rheumatoid arthritis and Alzheimer's disease may both be accompanied by IL1A polymorphisms^42^, ^43^. Immune cells play an important role in various immune responses in the human body, and gout, as an autoinflammatory condition, has been proven to be related to the immune system^44^. Therefore, on the Sangerbox website we performed an immunoassay for IL1A and detected a significant positive correlation of IL1A with B cells and a significant negative correlation with macrophages. B cells can mediate the inflammatory response in the pathogenesis of gout by synthesizing antibodies, and promote the accumulation of neutrophils in diseased tissues^45^, ^46^. In contrast, macrophages can switch to a pro-inflammatory state in the advanced stages of gout, increasing the inflammatory response of diseased tissues^47^. These two types of cells both play a role in the treatment of gout, which may help us further study the pathogenesis of gout from the perspective of immune cells. In addition, the ROC curve also showed that IL1A has significant clinical diagnostic value for clinical gout patients. And in PBMCs, the expression level of IL1A in gout patients was higher than that in normal healthy group.

We learned that the deposition of MSU crystals in joints triggers an acute inflammatory response^48^, ^49^. IL-8, IL-1β, TNFα are pro-inflammatory cytokines produced by M1 macrophages, and their high expression promotes the development of gout^50^. In addition, other studies have shown that TLR4 can mediate the release of pro-inflammatory cytokines through the MyD88 signaling pathway^51^. In experimental studies, treatment of cells with high concentrations of MSU resulted in up-regulated levels of IL1A, IL-1β, IL-8, and TNFα. When IL1A was knocked down, the expressions of the above cytokines decreased accordingly. Therefore, we conclude that IL1A promotes the development of gout by up-regulating Toll-like receptor signaling pathway.

In conclusion, through bioinformatic analysis on GSE199950 dataset, we find that IL1A is a risk factor for gout patients, and it has a good clinical diagnostic value. Cell experiments showed that after knocking down IL1A, the levels of pro-inflammatory cytokines also decreased. Therefore, we conclude that IL1A may participate in the inflammatory response of gout through Toll-like receptor pathway. However, due to the limitations in our study, this conclusion needs to be confirmed further.

## Supplementary Material

Supplementary table 1.
