# Supplementary material for: IL1A regulates the inflammation in gout through the Toll-like receptors pathway
Source: Int J Med Sci. 2024 Jan 1;21(1):188–99. doi: 10.7150/ijms.88447 (PMC10750337; doi:10.7150/ijms.88447)
Supplement: Supplementary file 1 — Supplementary table 1. [file ijmsv21p0188s1.pdf]

supplementry Table 1

Characteristics of gout patients and healthy controls.

	Items			
	Case number	Gender	Age	Uric acid (μ mol/L)
**GOUT(N=10)**	1	Male	78	542
	2	Male	85	461
	3	Male	64	518
	4	Male	64	553
	5	Male	68	442
	6	Male	69	498
	7	Male	52	453
	8	Male	75	534
	9	Female	51	434
	10	Female	89	456
**HC(N=5)**	1	Male	75	343
	2	Male	69	163
	3	Male	81	232
	4	Female	35	302
	5	Female	42	285
